# A Meta-Analysis of Therapeutic Efficacy and Safety of Gabapentin in the Treatment of Postherpetic Neuralgia from Randomized Controlled Trials

**DOI:** 10.1155/2018/7474207

**Published:** 2018-07-04

**Authors:** Meng Zhang, Cun-Xiang Gao, Ke-Tao Ma, Li Li, Zhi-Gang Dai, Sheng Wang, Jun-Qiang Si

**Affiliations:** ^1^Department of Anesthesiology, First Affiliated Hospital, School of Medicine, Shihezi University, Shihezi 832002, China; ^2^Department of Urology, First Affiliated Hospital, School of Medicine, Shihezi University, Shihezi 832002, China; ^3^Electrophysiological Laboratory, Laboratory of Xinjiang Endemic and Ethnic Diseases, China; ^4^Department of Physiology, Medical College of Shihezi University, Shihezi, Xinjiang 832002, China

## Abstract

**Objective:**

The study aims to systematically evaluate the clinical effect of gabapentin in the treatment of postherpetic neuralgia (PHN).

**Method:**

Data were retrieved electronically from PubMed, Embase, CNKI, the China Biomedical Database, and the Library of Clinical Database, beginning from the time of inception to April 2017, in order to collect data on randomized controlled trials (RCTs) of gabapentin and placebo in PHN treatment.

**Results:**

A total of 11 RCTs (2376 people) were retrieved. The gabapentin group reported significantly reduced pain intensity [MD=−0.91, 95% CI −1.32 to −0.51, P<0.00001] compared with the placebo group. Those treated with gabapentin also experienced significantly improved sleep quality [SMD=−0.44, 95% CI −0.66 to −0.23, P<0.0001], but were more likely to experience incidence of adverse events, such as somnolence, dizziness, and peripheral edema. Results of the subgroup analysis showed that the source of heterogeneity may be related to the formulations of the drug.

**Conclusion:**

Gabapentin can be used to effectively and safely treat PHN.

## 1. Introduction

Postherpetic neuralgia (PHN) is a sensory nervous system injury-based neuropathic pain, caused by the herpes zoster virus. PHN is a persistent burning and paroxysmal stimulation pain that lasts from several months to several years. It commonly occurs in the chest and back, but may also affect the whole body [1]. The pathogenesis of PHN has yet to be fully clarified, but most studies suggest that the herpes zoster virus located in the dorsal root ganglion is reactivated in people in old age (age ≥60 years, especially ≥80 years) or people with low immunity (e.g., cachexia and cancer patients), thereby leading to the degeneration of the spinal nerve sensory system and increased neuropathic pain [1–3].

At present, most of the clinical applications on neuropathic pain patients consisted of the antiviral treatment of early-onset herpes zoster. For patients with persistent pain, a combination of multiple medications is used, including opioid analgesics (morphine and oxycodone [4]), TCAs (amitriptyline [5] and doxepin hydrochloride), anticonvulsant [6] (gabapentin, pregabalin, and carbamazepine), topical drugs (piroxicam patch, lidocaine patch [7], and low concentrations of capsaicin patches), intrathecally given drugs (methylprednisolone [8]), and intravenous drugs (ketamine). All of these drugs have achieved good results to some extent, but their individual differences are large and cause many adverse reactions.

Gabapentin, associated with PHN, was initially used as an antiepileptic drug. It possesses a central analgesic effect and also inhibits the ectopic discharge of the peripheral nerve after injury [9]. Its major pharmacological mechanism is to block the Ca2 + channel *α*2*δ*-1 subunit and reduce the Ca2 + influx, thereby reducing the excitatory amino acid and excitatory neurotransmitter release [10, 11]. Its most common adverse effects are dizziness, somnolence, and peripheral edema. The most serious adverse effect is convulsion, which can also cause cognitive impairment among the elderly. Gabapentin also aggravates gait abnormalities and increases the risk of cardiovascular disease [12].

Some randomized controlled trials (RCTs) [13–21] have shown that gabapentin can effectively relieve pain in patients with PHN, while other studies [22, 23] have shown no significant difference in the efficacy and safety of gabapentin compared with placebo. Therefore, the efficacy and safety of gabapentin should be evaluated comprehensively. A meta-analysis of randomized, placebo-controlled clinical trials is conducted in this study to provide a complete and novel guide for the treatment of PHN using gabapentin.

## 2. Materials and Methods

### 2.1. Search Strategy

Data were retrieved electronically from PubMed, Embase, CNKI, the China Biomedical Database, and the Library of Clinical Database. Retrieval time began from inception to April 2017. The key words used were “gabapentin” and “postherpetic neuralgia.” The inclusion criteria from the study reference were used to retrieve the literature. The studies screened independently according to the standards of two reviewers.

### 2.2. Inclusion and Exclusion Criteria

The inclusion criteria were as follows: (1) study: RCTs, (2) research object: PHN patients, and (3) intervention measures: unlimited dosage of oral gabapentin or placebo. The exclusion criteria were as follows: (1) Non-RCTS, (2) study of poor balance between groups, (3) in vitro or animal trial, (4) use of other analgesics in addition to the conventional treatment, and (5) patients with other diseases, such as diabetes or AIDS, which might affect the treatment.

### 2.3. Evaluation of Trial Quality

The Jadad Standard was used to evaluate the quality of included studies. (1) Randomization grouping: random sequence by random number table or computer (2 points); the tests are randomly assigned, but the methods for generating random sequences are not accounted for (1 point) quasi-random or semirandom trials (0 point); (2) randomization concealment: appropriate (2 points), unclear (1 point), and unused or inappropriate (0 point); (3) blind: appropriate (2 points), unclear (1 point), and inappropriate (0 point); (4) exit: described reasons and number of exits (1 point), reasons and number of exits are not described (0 point).

### 2.4. Outcome Measures

The primary outcomes were as follows: pain intensity (VAS or NRS), the influence degree of pain on sleep, and the most common incidence of adverse events (somnolence, dizziness, and peripheral edema).

### 2.5. Statistical Analysis

Revman 5.3 software was used for the screening and meta-analysis of data. The data included the Chi^2^ test for heterogeneity and used I^2^ for quantitative analysis (test level 50%). If the analysis results show that P>0.05, I^2^≤50%, then the fixed effect model was used for meta-analysis. If the analysis results show that P<0.05, I^2^≥50%, then the random effect model was used for meta-analysis. When the data units of measurement are inconsistent or the measurement scales are different, the standardized mean difference (SMD) values were used instead of MD, and the effect was represented by 95% confidence interval (CI).

## 3. Results

### 3.1. Characteristics of Included Studies

**Figure 1:**
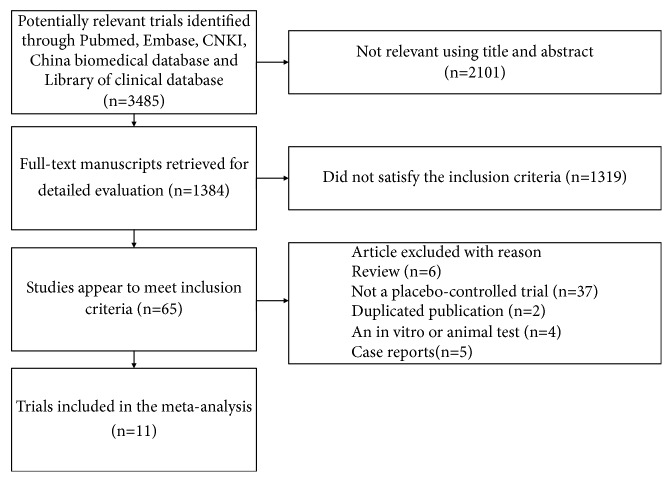
Flowchart of the trial selection for meta-analysis.

A total of 3485 relevant publications were identified by the initial electronic search. After reviewing the articles, 3474 studies were excluded, mainly because they were case reports or reviews or did not satisfy the inclusion criteria. Only 11 trials [13–23] were included in this meta-analysis (Figure 1).

The trials involved 2376 participants, of whom 1424 were assigned to the gabapentin group and 952 to the placebo group. Most studies were conducted in the USA [13, 16, 17, 22, 23] and China [18–21], and two [14, 15] were completed in the UK and France, respectively. Other details of included studies are shown in Table 1.

### 3.2. Gabapentin Efficacy

Gabapentin was used to treat PHN. Gabapentin has three formulations: Gabapentin, gabapentin ER, and GEn. Gabapentin ER was usually taken 1800 mg/day [13, 16, 22]. GEn had three administration methods: 1200, 2400, and 3600 mg/day [14, 23]. Gabapentin was given in the following doses: 1200, 1800, 2400, and 3600 mg/day [15, 17–21]. In the treatment of PHN, the use of different doses and different frequencies can produce different pharmacological effects. Therefore, the systematic evaluation of the efficacy and safety of different formulations and doses of gabapentin is critical.

Although the race, age, gender, frequency, and duration of drug administration may influence the outcome of the subgroup analysis of gabapentin, research about this matter is scarce. Instead, a subgroup analysis on the formulation of the drug (gabapentin ER, GEn, and gabapentin) was carried out.

### 3.3. Change in Average Daily Pain Score from Baseline

**Figure 2:**
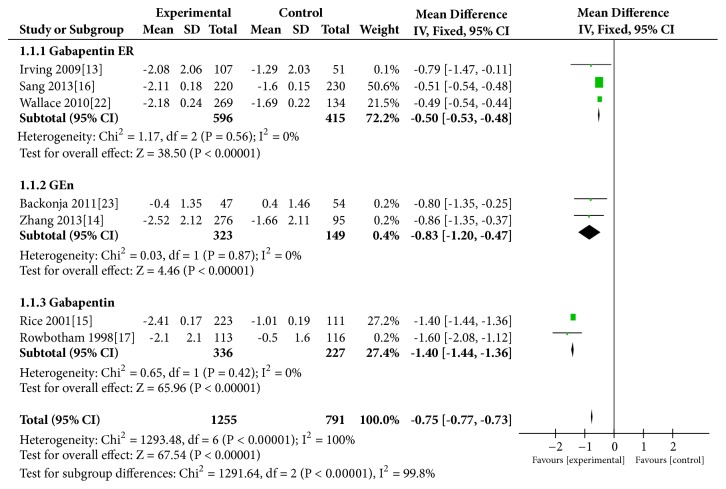
Change in average daily pain score from the baseline.

Seven [13–17, 22, 23] studies provided data about change in average daily pain score from the baseline, three [13, 15, 16] of which were measured using the Enguage software, and the rest came from the data in the tables. Compared with the placebo group, the gabapentin group changed more obviously (REM: MD=−0.91, 95% CI −1.32 to −0.51, P<0.00001; heterogeneity: I^2^=100%, P<0.00001; FEM: MD=–0.75, 95% CI −0.77 to −0.73, P<0.00001; heterogeneity: I^2^=100%, P<0.00001). The subgroup analysis based on drug formulation showed a similar trend among the gabapentin ER group (MD=−0.50, P<0.00001), GEn group (MD=−0.83,P<0.00001), and gabapentin group (MD=−1.40, P<0.00001). No heterogeneity (I^2^ = 0%) in the included trials was observed in the subgroup analysis (Figure 2).

### 3.4. At Least 50% Reduction in Pain Intensity

**Figure 3:**
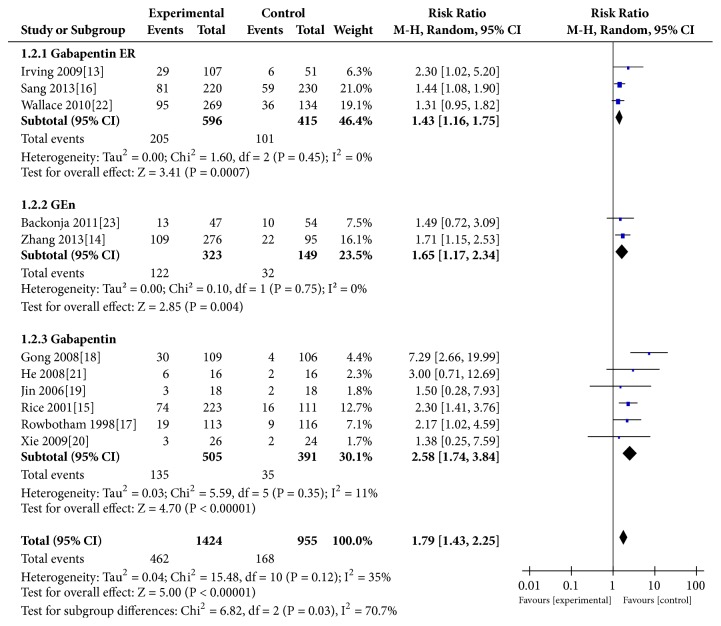
At least 50% reduction in pain intensity.

All the studies [13–23] showed that the gabapentin group was significantly better than the control group (REM:RR=1.79, 95% CI 1.43 to 2.25, P<0.00001; FEM:RR=1.75, 95% CI 1.50 to 2.05, P<0.00001; heterogeneity: P=0.12, I^2^=35%). The subgroup analysis showed a similar trend about this endpoint among the gabapentin ER group (RR=1.45, P=0.0004), GEn group (RR=1.66, P=0.004), and gabapentin group (RR=2.72, P<0.00001) (Figure 3).

### 3.5. Reduction in Sleep Rating Scores

**Figure 4:**
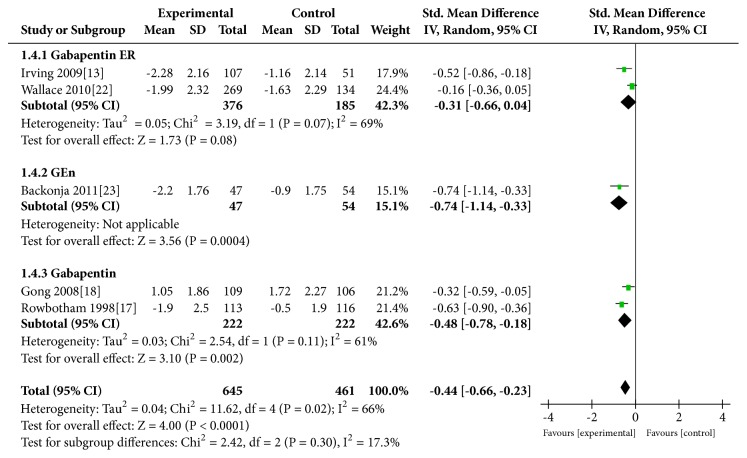
Reduction in sleep rating scores.

Five [13, 17, 18, 22, 23] trials evaluated the reduction in sleep rating scores.  Figure 4 shows that the gabapentin group showed a significant improvement in sleep rating scores compared with the placebo group. (REM: SMD=−0.44, 95% CI −0.66 to −0.23, P<0.0001; heterogeneity: I^2^=66%, P<0.0001; FEM: SMD=−0.39, 95% CI −0.52 to −0.27, P<0.00001; heterogeneity: I^2^=66%, P<0.00001). The subgroup analysis based on drug formulation showed a similar trend among the gabapentin ER group (SMD=−0.31, P=0.08), GEn group (SMD=−0.74, P=0.0004), and gabapentin group (SMD=−0.48, P=0.02) (Figure 4).

### 3.6. Patient Global Impression of Change (PGIC)

**Figure 5:**
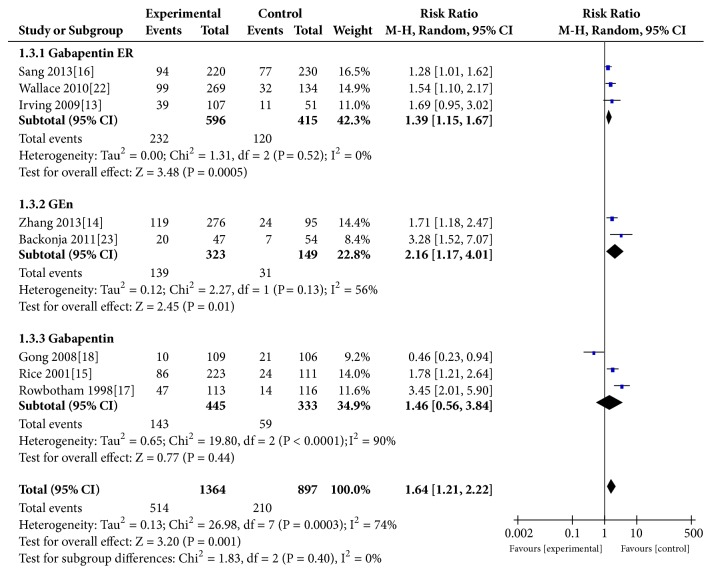
PGIC.

The effect model meta-analysis of data from eight [13–18, 22, 23] studies showedthat the proportion of patients with this result was higher in thegabapentin group than in the placebo group (REM:RR=1.64, 95% CI 1.21 to 2.22, P=0.001; heterogeneity, P=0.0003, I^2^=74%; FEM: RR=1.59, 95% CI 1.38 to 1.82, P<0.00001). The subgroup analysis of this endpoint showed a similar trend among the gabapentin ER group (RR=1.39, P=0.0005), GEn group (RR=2.16,P=0.01), and gabapentin group (RR=1.46, P=0.44) (Figure 5).

### 3.7. Adverse Event

**Figure 6:**
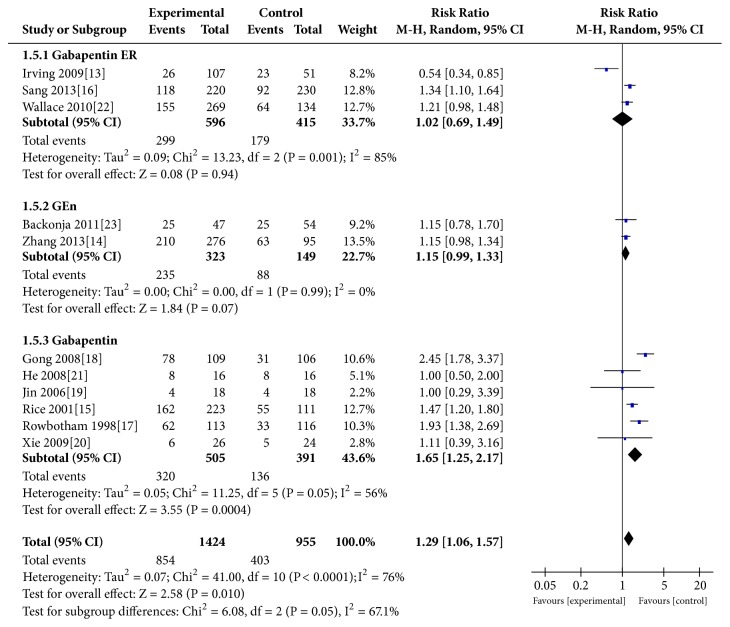
Adverse event.

All included studies [13–23] reported the relationship between the occurrence of adverse event and gabapentin formulations. Despite the differences in dosage, gabapentin significantly increased the risk of adverse event (REM:RR=1.29,95% CI 1.06 to 1.57, P=0.010; heterogeneity, P<0.0001, I^2^=76%; FEM:RR=1.34,95% CI 1.23 to 1.46, P<0.00001). The subgroup analysis based on formulations was also performed with the following results: gabapentin ER group (RR=1.02, P=0.94), GEn group (RR=1.15, P=0.07), and gabapentin group (RR=1.65, P=0.0004) (Figure 6).

## 4. Discussion

Our main results (Figures 2, 3, and 5) indicated that, compared with the placebo group, the gabapentin groups showed more significant advantages in the treatment of PHN patients, and patients who received gabapentin treatment may experience less exit. At the same time, gabapentin had some side effects, such as peripheral edema, somnolence, and dizziness, which, statistically speaking, showed a significant increase in the treatment process (P=0.010) (Figure 6). In fact, the results of the subgroup analysis of formulations showed that, compared with the placebo group, the differences in the incidence side effects of the gabapentin ER group (p=0.94) and GEn group (p=0.07) were not statistically significant.

According to our inclusion criteria, the quality of research is very high (Jadad ≥4); only four researches [16, 20, 21, 23] did not describe the reason and the number of exit. All of the researches had a randomized, double-blind, parallel, and placebo experimental design, which, to a certain extent, may increase the relative risk of adverse events in the placebo group.

The results of the sensitivity analysis by changing the effect mode show that most of the results are relatively stable in addition to a small number of patients because of the side effect withdrawal of drug. If the analysis results show that P>0.05, I^2^ ≥50%, then the fixed effect model can be used for meta-analysis; if the analysis results show that P<0.05, I^2^ ≤50%, then the random effect model can be used for meta-analysis. The subgroup analysis can be used to analyze the sources of heterogeneity. If the measurement unit of data is inconsistent, then the SMD value is used to replace MD. The subgroup analysis showed that the sources of heterogeneity may be related to the formulations of gabapentin. In addition, the GEn group performed better than other drug formulations, indicating that its formulation is considerably safe. However, this conclusion should be demonstrated further, especially through the control of the gabapentin dosage administered to patients.

In addition, [22] was contrary to our meta-analysis result: the ADP changes of the gabapentin group compared with the placebo group had no significant difference (Gabapentin ER1800mg/d, divided dose). The authors of that study emphasized that the phenomenon of spontaneous remission in patients with PHN and the course of disease duration may be important confounding factors. In our study, no detailed data of patients with the duration of PHN exist, making it difficult to assess whether this factor affected our results. Thus, such a factor should be considered in future research.

Moreover, Moore et al.[17] suggested that the gabapentin group exhibited a significant benefit in the treatment of PHN, compared with the placebo group, and the result is consistent with our analysis. However, some of their research and our analysis results are different. First, the primary outcomes are evaluated but the evidence is relatively weak (change in average daily pain score from baseline, at least in pain intensity and 50% reduction and PGIC), thus requiring more rigorous tests in future research. Second, our analysis includes three pharmaceutical formulations, including gabapentin, gabapentin ER, and GEn; hence, our research was more comprehensive. Third, our analysis focused on a single type of neuropathic pain, PHN, to obtain uniform and stable results. Moore et al. collected various types of neuropathic pain and fibromyalgia. Fourth, a subgroup analysis for gabapentin formulations was conducted, and the results showed the efficacy and safety of GEn. Fifth, the reduction in sleep rating scores of patients with PHN was evaluated, and the index in the study of Moore et al. was not analyzed.

## 5. Conclusions and Limitations of the Study

Gabapentin can relieve pain in PHN significantly. To maximize the efficacy and reduce adverse events, the appropriate formulations of the drug should be thoroughly considered. The GEn group showed better results than the other groups, indicating that this is better than the other formulations in the treatment of PHN effectively and safely. However, the long-term clinical efficacy and safety in different formulations of gabapentin remains to be determined.

Nevertheless, our study has limitations. First, most of our references are English publications; therefore, we may have missed miss nonmainstream and gray research. Second, most of the studies are from the United States; hence, most of the participants are White, thereby limiting our research results to other races. Third, the long-term safety and efficacy of gabapentin therapy in PHN cannot be assessed because the studies included are only for short-term treatment regimes. Fourth, four studies [13, 15, 16, 22] are included because of ethical reasons. Patients who have a low tolerance of gabapentin are excluded, which may lead to the overestimation of the clinical effect of gabapentin.

Overall, our study shows that, compared with the placebo, gabapentin can significantly relieve pain in PHN patients and reduce the pain of sleep disorders. Moreover, PHN patients have good tolerance to gabapentin. Future research should explore the different dosages, durations, and frequencies of gabapentin administration in the treatment of PHN. These studies should also examine the therapeutic effect and safety of gabapentin when administered to different races. Finally, long-term follow-up should be included.

## Figures and Tables

**Table 1:** Summary of clinical trials included in meta-analysis.

Study	Design	Intervention (sample size and age)	Duration (week)	Primary endpoints	Secondary endpoints	Common AEs	Jadad score
Irving 2009 [13]	Double-blind, randomized, placebo-controlled, parallel	158 included in the ITT analysis: Gabapentin ER1800 mg, QD, PM (55) (71±10.3); BID, 600 mg AM, 1200 mg PM (52) (68±12.9) placebo (51) (69±11.5)	5	ADP scores	*⩾*30% improvement, *⩾*50% improvement, Sleep interference, SF-MPQ, NPS, PGIC/CGIC	Dizziness, Somnolence, Nausea, Dry mouth, Headache, Fatigue, Gait disturbance, Peripheral edema, Upper respiratory tract infection	5
Wallace 2010 [22]	Double-blind, randomized, placebo-controlled, multicenter, parallel	400 included in ITT analysis: Gabapentin ER 1800 mg, QD (134) (68±11.8); BID (135) (66±13.2) placebo (131) (66±12.6)	11	ADP scores	*⩾*50% improvement, Sleep interference, SF-MPQ, NPS, BPI, PGIC/CGIC	Dizziness, Headache, Somnolence, Peripheral edema	5
Backonja 2011 [23]	Double-blind, randomized, placebo-controlled, parallel	101 included in ITT analysis: GEn1200 mg, BID (47) (65±12.32); placebo (54)(64±12.69)	4	ADP scores	*⩾*30% improvement, *⩾*50% improvement, Sleep interference, POMS, PGIC, SF-MPQ	Dizziness, Nausea, Headache, Diarrhea, Fatigue, PHN, Insomnia, Depression	4
Zhang 2013 [14]	Double-blind, randomized, placebo-controlled, multicenter, parallel	371 included in the ITT analysis: GEn 1200 mg, BID, (107) (61.7±12.58); GEn 2400 mg, BID, (82) (64.1±8.94); GEn 3600 mg, BID, (87) (61.3±15.41); Placebo (95) (61.7±12.77)	14	ADP scores	*⩾*30% improvement, *⩾*50% improvement, Pain intensity, Sleep interference, NPS, SF-MPQ, BPI, SF-36, Dynamic Allodynia, POMS-B, PGIC/CGIC, Use of Rescue Medication	Dizziness, Somnolence, Headache, Nausea, Constipation, Diarrhea, Fatigue, Nasopharyngitis, Edema peripheral, Arthralgia, Insomnia, Urinary tract infection, Back pain, Weight increase, Dry mouth, Hypertension, Nasal congestion, Vision blurred, Flatulence, Joint sprain, Tremor	5
Rice 2001 [15]	Double-blind, randomized, placebo-controlled, multicenter, parallel	334 included in ITT analysis: Gabapentin 1800 mg (115) (74.8, 22.5–88.6); 2400 mg (108) (76.3, 36.1–90.8); Placebo (111) (74.9, 28.9–94.8)	7	ADP scores	*⩾*30% improvement, *⩾*50% improvement, Sleep interference, SF-MPQ, PGIC/CGIC, SF-36	Dizziness, Somnolence, Peripheral edema, Asthenia, Dry mouth, Diarrhea	5
Sang 2013 [16]	Double-blind, randomized, placebo-controlled, multicenter, parallel	450 included in the ITT analysis: gabapentin ER 1800 mg, QD (220) (65.3±13.3); Placebo (230) (65.9±11.1)	10	ADP scores	*⩾* 50% improvement, PGIC/CGIC, Sleep interference	Dizziness, Somnolence, Headache, Nausea, Peripheral edema, Nasopharyngitis	4
Rowbotham 1998 [17]	Double-blind, randomized, placebo-controlled, multicenter, parallel	229 included in the ITT analysis: Gabapentin 3600 mg, TID (113) (73,40–90); placebo (116)(74,39-89)	8	ADP scores	Sleep interference, SF-MPQ, PGIC/CGIC, SF-36, POMS	Somnolence, Dizziness, Ataxia, Peripheral edema, Infection	5
Gong 2008 [18]	Double-blind, randomized, placebo-controlled, multicenter, parallel	215 included in the ITT analysis: Gabapentin divided-doses 1800 mg (109) (67.49±11.55); Placebo (106) (65.25±12.11)	6	ADP scores	*⩾* 50% improvement, ADP,SF-MPQ,PGIC/CGIC	Dizziness, Somnolence, Amblyopia, Peripheral edema, Nausea, Vomiting, Nystaxis, Dysuria	5
Jin 2006 [19]	Double-blind, randomized, placebo-controlled, parallel	36 included in the ITT analysis: Gabapentin divided-does 2400 mg (18) (63±10.3); Placebo (18) (64.39±9.12)	4	ADP scores	*⩾* 50% improvement, ADP, PGIC/CGIC	Dizziness, Somnolence, Ataxia, Nausea, Vomiting	5
Xie 2009 [20]	Double-blind, randomized, placebo-controlled, parallel	50 included in the ITT analysis: Gabapentin divided-does 2400 mg (26); Placebo (24)	4	ADP scores	*⩾* 51% improvement, ADP, PGIC/CGIC	Dizziness, Somnolence, Anorexia, Insomnia	4
He 2008 [21]	Double-blind, randomized, placebo-controlled, parallel	32 included in the ITT analysis: Gabapentin divided-does 1200 mg (16) (74.1±12.2); Placebo (16) (72.69±11.7)	4	ADP scores	*⩾* 52% improvement, ADP, PGIC/CGIC,QS	Dizziness, Somnolence, Week, Ataxia, Anorexia, Insomnia, Vertigo	4

ITT: intent to treat; GEn: gabapentin enacarbil; gabapentin ER: gabapentin extended-release; QD: once daily; BID: twice daily; TID: three times daily; ADP: average daily pain scores; SF-MPQ: Short Form-McGill Pain Questionnaire; NPS: Neuropathic Pain Scale; PGIC/ CGIC: Patient/Clinical Global Impression of Change; BPI: The Brief Pain Inventory; POMS: the Profile of Mood States; SF-36: Short Form-36; QS: Quality Of Sleep.
